# Prevalence of *von Hippel-Lindau *gene mutations in sporadic renal cell carcinoma: results from the Netherlands cohort study

**DOI:** 10.1186/1471-2407-5-57

**Published:** 2005-06-02

**Authors:** Kjeld P van Houwelingen, Boukje AC van Dijk, Christina A Hulsbergen-van de Kaa, Leo J Schouten, Hanneke JM Gorissen, Jack A Schalken, Piet A van den Brandt, Egbert Oosterwijk

**Affiliations:** 1 Department of Urology, University Medical Center Nijmegen, P.O. Box 9101, 6500 HB Nijmegen, the Netherlands; 2 Department of Epidemiology, NUTRIM, Maastricht University, P.O. Box 616, 6200 MD Maastricht, the Netherlands; 3 Department of Pathology, University Medical Center Nijmegen, P.O. Box 9101, 6500 HB Nijmegen, the Netherlands

## Abstract

**Background:**

Biallelic *von Hippel-Lindau *(*VHL*) gene defects, a rate-limiting event in the carcinogenesis, occur in approximately 75% of sporadic clear-cell Renal Cell Carcinoma (RCC). We studied the *VHL *mutation status in a large population-based case group.

**Methods:**

Cases were identified within the Netherlands cohort study on diet and cancer, which includes 120,852 men and women. After 11.3 years of follow-up, 337 incident cases with histologically confirmed epithelial cancers were identified. DNA was isolated from paraffin material collected from 51 pathology laboratories and revised by one pathologist, leaving material from 235 cases. *VHL *mutational status was assessed by SSCP followed by direct sequencing, after testing SSCP as a screening tool in a subsample.

**Results:**

The number of mutations was significantly higher for clear-cell RCC compared to other histological types. We observed 131 mutations in 114 out of 187 patients (61%) with clear-cell RCC. The majority of mutations were truncating mutations (47%). The mean tumor size was 72.7 mm for mutated tumors compared to 65.3 mm for wildtype tumors (p = 0.06). No statistically significant differences were observed for nuclear grade, TNM distribution or stage. In other histological types, we observed 8 mutations in 7 out of 48 patients (15%), 1 mutation in 1 of 6 oncocytoma, 3 mutations in 2 of 7 chromophobe RCC, 2 mutations in 2 of 30 papillary RCC, no mutations in 1 collecting duct carcinoma and 2 mutations in 2 of 4 unclassified RCC.

**Conclusion:**

*VHL *mutations were detected in 61% of sporadic clear-cell RCC. *VHL *mutated and wildtype clear-cell RCC did not differ with respect to most parameters.

## Background

Historically, the classification of Renal Cell Cancer (RCC) was based on morphological features. The majority of RCC are of the clear cell type (~80%); other subtypes are papillary RCC (10%), chromophobe RRC (5%), collecting-duct carcinoma (1%) and unclassified RCC (3–5%). Based on the work of numerous investigators, it became evident that RCC could be divided into genetically distinct classes: this resulted in the so-called Heidelberg classification, which partly overlaps the former pathological classification of RCC based on morphological criteria. The most prominent common genetic aberration for clear-cell (conventional) RCC is loss of 3p. Characteristic for papillary RCC is trisomy of chromosomes 3q, 7,8,12,16,17,20, and loss of the Y chromosome, and chromophobe RCC is characterized by a combination of loss of heterozygosity at chromosomes 1,2,6,10,13,17, and 21 [1].

Von Hippel-Lindau disease (VHL) is a rare inherited disorder associated with, amongst others, an enhanced risk for clear-cell RCC [2]. The *VHL *gene responsible for this syndrome was identified through linkage analyses and molecular cloning and is located on chromosome 3p25. After its identification it became evident that the *VHL *gene is also involved in the development of sporadic clear-cell RCC. Together with loss of the homologous chromosome 3p allele (3p LOH), *VHL *mutations are rate-limiting events in the carcinogenesis of clear-cell RCC [3,4]. Mutations have been observed in the entire gene and usually lead to a truncated inactive protein [5]. The VHL gene is considered a tumor suppressor gene, involved in cell cycle regulation, regulation of hypoxia inducible genes and proper fibronectin assembly in extracellular matrix [6,7]. It was estimated that approximately 75% of all sporadic clear-cell RCC harbor biallelic *VHL *defects [8]. In approximately 19% of sporadic clear-cell RCC, methylation of the *VHL *gene promoter appeared to be involved [9]. In approximately 10%–20% of sporadic clear-cell RCC no alteration in the *VHL *alleles was detected, indicating that other genes are involved in clear-cell RCC carcinogenesis, possibly affecting the same signaling pathway as VHL.

Several risk factors for developing RCC have been identified: tobacco smoking, obesity, drugs, such as phenacetin, hypertension and/or its medication, and occupational exposure to trichloroethylene, gasoline, petroleum products, asbestos, and iron processing fumes. The influence of dietary factors, such as vegetable, fruit vitamin C, carotenoid, meat and milk product consumption, is controversial [10]. Multiple and specific types of *VHL *mutations in RCC have been associated with exposure to the industrial solvent trichloroethylene [11,12]. Consumption of vegetables and citrus fruit decreased the frequency of *VHL *mutations among smokers and consumption of citrus fruit decreased *VHL *mutation frequency for all patients [13]. These findings and investigations in animals [14] suggest that mutational patterns in the *VHL *gene may serve as an etiological imprint to factors causing renal cancer. Thus, it may be possible to improve our etiological insight in particular risk factors when a more specific endpoint than "RCC" can be defined, e.g. based on histology and mutational status of a gene involved in tumor carcinogenesis.

We decided to determine the mutational status of the *VHL *gene of RCC cases identified within a population-based cohort of 120,852 men and women aged 55–69 which was recruited in the Netherlands to study associations between dietary habits, lifestyle and cancer occurrence. To validate whether SSCP could serve as a prescreening method, SSCP and direct sequencing was evaluated in a subset of 20 patients. In this article we report on histopathological and clinical parameters and the type and number of mutations in *VHL *observed in a large population-based sample of cases.

## Methods

### Study population

The RCC cases selected for this study were incident cases identified in the population of the Netherlands Cohort Study (NLCS) on diet and cancer. The NLCS has been described in detail elsewhere [15]. Briefly, this prospective study was initiated in 1986 and included 58,279 men and 62,573 women, 55 to 69 years of age, who completed a self-administered questionnaire on diet, family history of cancer, and other risk factors for cancer at baseline. The entire cohort is being monitored for cancer occurrence by annual record linkage to the Netherlands Cancer Registry and to PALGA, a nationwide network and registry of histo- and cytopathology [16]. Information on sex and family history of RCC (at baseline) was retrieved from the NLCS database, while information on age at diagnosis and tumor localization was retrieved from the Netherlands Cancer Registry. Tumor stage was classified according to the 1987 revision of the UICC-TNM classification [17], using information from the cancer registry and the pathology report. Tumor size was retrieved from the pathology report.

### Tissue sample collection

Paraffin material was collected after approval by the Medical Ethical Committees of Maastricht University, PALGA and the Netherlands cancer registry. From 1986 to 1997 (11.3 years follow up) 355 kidney cancer cases (ICD-O-3: C64.9) were identified. Urothelial cell carcinomas were excluded and only histologically confirmed epithelial cancers were included (ICD-O: M8010-8119, 8140–8570), leaving 337 cases. The PALGA database was also used to identify the location of tumor tissue storage in the Dutch pathology laboratories. For 273 cases, a PALGA record with information on the location of paraffin material was available at the start of the collection of paraffin blocks. The tissue samples were distributed over 51 pathology laboratories throughout the Netherlands.

For 251/273 cases (92%) paraffin blocks were collected. Failure to retrieve material was the result of the refusal of the pathology laboratory to cooperate (3 laboratories with material for 10 cases), the unavailability of suitable material (i.e. only material from a biopsy, cytology or a metastasis was present) (8 cases), not being able to locate the paraffin block at the laboratory (3 cases), and for 1 case the reason was not recorded. Paraffin blocks of parallel normal tissue were collected if available. Collected archival tissue sample blocks were registered and coded using a unique identification number.

### Revision

Genomic DNA was extracted from five 20-μm slices from each tumor and normal specimen. A flanking section was haematoxylin and eosin (HE) stained for histological purposes, e.g. grading and estimation of percentage of tumor cells. One experienced pathologist (CAHK) reviewed all HE stained slides. The RCC were classified according to the World Health Organization (WHO) classification of Tumours of 2002 [18]. Nuclear grading was performed according to Fuhrman [19]. Grading was based on the most atypical focus in the paraffin block used for DNA extraction, with a dimension of at least one high power field.

Material of 16 cases was discarded after revision. The collected material was unsuitable for analysis, because it concerned a biopsy (N = 2) or a metastasis (N = 2) or no tumor tissue was present (N = 4) or material contained less than 10% malignant cells (N = 7) (tumor samples had to contain at least 10% malignant cells to decrease the possibility of ignoring mutations). Material from one case was reclassified as urothelial cell carcinoma and subsequently excluded. As a result, tumor DNA from 235 cases was available for further analysis.

### DNA isolation

Genomic DNA was prepared as follows: paraffin was removed with xylene and genomic DNA was extracted by salt-precipitation. Briefly, 450 μl of cell lysis solution (10 mM Tris/HCl (pH 7.4), 400 mM NaCl, 2 mM EDTA), 25 μl of 10% SDS and 50 μl of proteinase K solution (20 mg/ml) were added to the tissue samples and incubated over-night at 55°C. Proteins were precipitated using 175 μl of saturated NaCl, followed by centrifugation (2 minutes, 13.200 rpm). DNA was precipitated by the addition of 0.6 volumes of iso-propanol, dissolved in TE (pH 7.4) and stored at -20°C. The DNA concentration and purity was measured at 260 and 280 nm.

### *VHL *mutation analysis

PCR primers used for amplification are described in Table 1. Amplification of DNA for SSCP analysis and sequence analysis was performed as follows: 100 ng of the extracted DNA was subjected to 35 cycles of PCR: 40 sec at 92°C, 40 sec at Tm and 40 sec at 72°C. Exon amplification was performed in 30 μl of buffer: 50 mM KCl, 10 mM Tris-HCl (pH 9.0), 1.5 mM MgCl2, 1% triton X-100, 0.1% (w/v) gelatin, 250 uM dNTP's, 25 pmol of each primer and 1U Taq polymerase (HT Biotechnology Ltd.). For SSCP analysis, 0.2 μl of [α 32P] dATP (10 mCi/ml, ~3000 Ci/mmol, FIRMA) was added.

For SSCP analyses, 5 μl of the radiolabeled PCR product was diluted in 5 μl loading buffer (96% formamide, 20 mM EDTA, 0.05% bromophenol blue and xylene cyanol), boiled for 3 min, and quenched on ice before loading. Three μl of each sample was loaded on a 0.5x MDE gel, containing 0.6x TBE either with or without 10% (v/v) glycerol. Electrophoresis was performed at room temperature at 5W for MDE gels without glycerol, and at 7W for MDE gels with 10% (v/v) glycerol, using 0.6x TBE as electrophoresis buffer. After 20 hours of electrophoresis, the gels were transferred to Whatmann 3 MM paper (FIRMA) and dried on a gel dryer. The separated fragments were visualized by Hyperfilm-MP (FIRMA) exposure.

For sequence analysis, the PCR products were purified using the Wizard™ PCR preps purification system (Promega Corp.). Sequencing was performed at the local central sequence facility using BigDye Terminator and the ABI basecaller (Applied Biosystems). Mutations were identified by visual inspection of sequences provided by the ABI basecaller, and called when unequivocally present on the sense and/or antisense strand, and when the area under the curve of peaks changed by more than 5%, compared to the area under the curve of the normal signal.

### Feasibility and comparability pilot

Twenty samples were analyzed by PCR-SSCP and direct sequencing to investigate the suitability of PCR-SSCP as a screening tool preceding direct sequencing. After evaluation, we decided to use PCR-SSCP as a screening instrument before direct sequencing. All remaining samples were subjected to PCR-SSCP, which was only followed by direct sequencing in case of an aberrant band pattern on the PCR-SSCP. Samples were also sequenced when equivocal PCR-SSCP results were obtained.

### Statistical analyses

The overall frequency of *VHL *mutations as well as the type of mutation and affected exon and codon were computed for all 235 cases. Differences between histological subtypes were assessed by the χ^2^-test. For clear-cell RCC, mutated and wildtype tumors were compared with respect to age at diagnosis, sex, grade, TNM classification [17], stage and family history of RCC. Differences in mean values of age at diagnosis as a continuous variable were evaluated by the student's t-test. Differences in the categorical variables sex, family history of RCC, grade, TNM classification and stage were evaluated for significance by the χ^2^-test. A p-value of 0.05 or less was considered statistically significant. Statistical analyses were performed with the STATA statistical software package (STATA statistical software, Release 7, STATA corporation, College Station, TX, USA, 2001).

## Results

### PCR-SSCP as a screening tool preceding direct sequencing

To evaluate whether PCR-SSCP could be used to distinguish between wildtype and mutated *VHL*, we determined the *VHL *status of 20 cases by SSCP and direct sequencing (Table 2). SSCP and direct sequencing were mostly in agreement. In all cases where disparate results were obtained, this concerned aberrant signals in PCR-SSCP, followed by a negative result by direct sequencing (Table 2). Most importantly, we never found a mutation in the sequence analysis after a negative PCR-SSCP (0% false-negatives) (Table 2). However, we are aware that the sampling size is limited, and it is known that SSCP as a screening tool is neither 100% sensitive nor specific. Therefore, we estimated the chance of a positive result on direct sequencing after a negative result on the SSCP (i.e., the chance that a mutation will be missed by using SSCP as a screening instrument). The point estimate for the percentage of false-negatives based on SSCP is 0%, with an upper confidence limit between 3.7% and 16.9%, based either on the individual analyses (N = 97), or on the number of cases (N = 20) (see Additional file 1: Calculation of the estimated upper 95% confidence limit). Different types of mutations were identified by SSCP followed by direct sequencing in this pilot.

### *VHL *mutations and clinical parameters

We were able to analyze at least one tumor block for 235 patients with 236 tumors (one patient had two primary tumors). After revision by CAHK, the analyzed samples could be subdivided as follows: clear-cell RCC (188/236, 79.7%), papillary RCC (30/236, 12.7%); chromophobe RCC (7/236, 3.0%); oncocytoma (6/236, 2.5%), collecting duct carcinoma (1/236, 0.4%), and unclassified RCC (4/236, 1.7%). For 198 cases one paraffin block was analyzed, while for 34 cases 2 paraffin blocks were analyzed and for 3 cases 3 paraffin blocks were analyzed. In 10 cases, a different genotype was observed in different tumor blocks obtained from 1 tumor. In 6 cases, 1 sample contained wildtype *VHL *while the other sample harbored one or more mutations. For the statistical analyses these cases were analyzed as "mutated". In 13 cases, 2 mutations in the same paraffin block were observed. In one patient with two primary tumors, both tumors showed a different mutation. In total we observed 253 outcomes (139 mutations (in 121 patients) and 114 wildtype) for 235 patients with 236 primary tumors. All 139 observed mutations are described. (see Additional file 2: Description of observed mutations (N = 139), histological parameters and personal characteristics for 121 cases).

*VHL *gene mutations were mostly observed in clear-cell RCC, but also in unclassified RCC (2/4); papillary RCC (2/30); chromophobe RCC (3 mutations in 2/7 patients) and oncocytoma (1/6) (see Additional file 2: Description of observed mutations (N = 139), histological parameters and personal characteristics for 121 cases). The percentage of patients with a mutation was significantly higher for clear-cell tumors compared to tumors of other histological types (χ^2^: 32.9; p-value<0.001). In the remainder of this paper, we will only consider clear-cell RCC, leaving 204 outcomes (131 mutations (in 114 patients) and 73 wildtype) for 187 patients with 188 primary tumors. Nine outcomes were mutations in intronic sequences, possibly affecting splicing. The majority of observed mutations in coding regions lead to a truncated VHL product (insertion/deletion mutations leading to a frameshift and nonsense mutations, 62/122) or to a deleted/inserted or altered amino acid, 47/122). The percentage of point mutations was much higher in exon 1 (50.7%) than in exon 2 and 3 (29.6 and 23.1, respectively).

**Figure 1:**
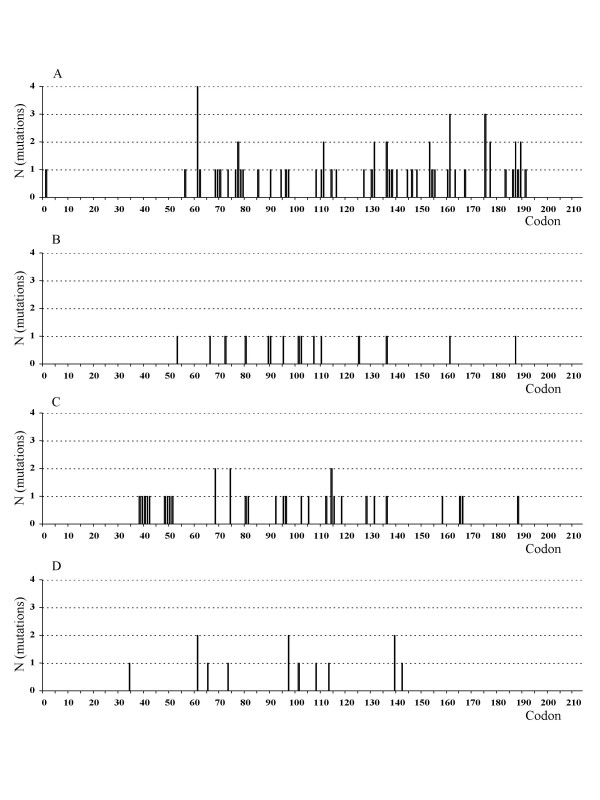
**Type of mutation plotted against the codon number. **A. Truncating mutations (Frameshift & Nonsense mutations) B. In-frame insertions/deletions C. Missense mutations D. Silent mutations Codon 1–114 encodes exon 1, codon 114–155 encode exon 2, and codon 155–213 encode exon 3.

Figure 1 shows the type of mutation plotted against the codon number. We observed 62 truncating mutations, which consisted of 4 nonsense mutations and 58 deletions or insertions leading to a shift of the reading frame (Figure 1A), 15 insertions or deletions that did not affect the reading frame (Figure 1B), 32 missense mutations (Figure 1C) and 13 silent mutations (Figure 1D).

Cases with a mutation were older (mean: 67.8 years; sd: 4.7) than cases without a mutation (mean: 67.1; sd: 4.6). The mean age difference at diagnosis was 0.8 years with a 95% CI ranging from -0.6 through 2.2 years. The percentage of men with clear-cell RCC with a *VHL *mutation was somewhat higher than the percentage of males among patients with a clear-cell RCC without a *VHL *mutation, 63.2 % vs. 53.4% (χ^2^: 1.75; *p*-value: 0.19).

The mean tumor size was 72.7 mm for tumors with a *VHL *mutation compared to 65.3 mm for tumors without a *VHL *mutation. The difference was 7.3 mm with a 95% CI from -2.1 mm through 16.8 mm. Other tumor parameters for patients with a primary clear-cell RCC with or without a *VHL *mutation are shown in Table 3. We did not observe a difference in nuclear grade, T, N, M, or stage between mutated and wildtype tumors.

For 3 patients, a positive family history was reported; 2 of these patients (Sample id's: 1003 and 306/1600) had a clear-cell tumor harboring a mutation. One patient with a papillary tumor reported a positive family history; no mutations in the *VHL *gene were observed in the corresponding tumor sample. Obviously, these numbers are too small to engage in statistical testing.

## Discussion

The *von Hippel-Lindau *(*VHL*) gene is a tumor suppressor gene predisposing to both sporadic clear-cell (conventional) RCC and von Hippel-Lindau disease. We analyzed 235 cases of primary sporadic RCC, of which 187 cases presented with the histological subtype clear-cell RCC, identified within the Netherlands cohort study on diet and cancer. *VHL *mutations were assessed by PCR-SSCP, followed by direct sequencing when aberrant signals were detected in SSCP. Screening by SSCP was considered appropriate, since we never detected a mutation by direct sequencing after a negative result on the SSCP in a pilot study. Furthermore, we estimated the upper 95% confidence limit for cases missed at 3.7% or 16.9%, based on the individual analyses (N = 97) or on the number of cases (N = 20). Therefore, mutated cases may have been overlooked, which placed them in the wildtype group. This would have resulted in a reduction of contrast between wildtype and mutated groups.

We observed 131 mutations in 114 out of 187 patients (61%) in patients with clear-cell tumors. Estimates reported in the literature [2,20-30] range from 36% [25] to 54% [27], and therefore our estimate appears high. However, our estimate is close to the estimate of 75% as discussed in the review by Cohen [8]. This difference in the observed proportion of mutations in the *VHL *gene may be attributed to the use of different techniques, e.g., SSCP vs. direct sequencing, to contamination with normal tissue components, or to the type of material analyzed (fresh or archival paraffin material). In our study, we used SSCP followed by direct sequencing, as most investigations did [2,20-29]. To avoid ignoring *VHL *mutations through contamination with normal tissue, samples needed to contain at least 10% tumor cells. The vast majority of samples (180 out of 190) contained at least 50% viable tumor tissue. The sample with the lowest percentage of viable tumor tissue, in which we observed a mutation, contained 30% of viable tumor tissue. For 2 samples the percentage of viable tumor tissue was lower (10% and 20%), thus we cannot formally exclude the possibility that we overlooked an existing mutation. We identified cases in a population-based study after 11.3 years of follow-up; the time span and the number of patients and hospitals involved excluded the possibility to collect fresh material. Three other studies on sporadic RCC used paraffin embedded material and the percentages of mutations detected were 46% [29], 50% [22] and 54% [27].

The occurrence of various types of mutations is comparable to other studies of this size [21,24,26,27], but some differences are also apparent. The presence of silent mutations is not commonly reported; only Ma et al. [27] reported 13% of silent mutations, similar to 11% observed by us. It is possible that others do not report silent mutations because the investigators do not wish to designate these as true mutations since their relevance is unclear. The percentage of frameshift mutations was approximately 50% in most studies [21,26], comparable to the 48% observed in the current study. The percentage of in-frame deletions/insertions was higher in our study (12%) as compared to the percentage in other studies (<5%) [21,26]. Compared to the distribution of mutation data present in the Universal *VHL *Mutation database with 747 mutations [31], the relative amount of point mutations is much higher in the database (64%), compared to the percentage of point mutations we observed (44%), as well as the frequency of nonsense mutations (11% vs. 3%). However, many of the mutations in the Mutation database concern germline mutations identified in VHL-families. It has been suggested that the mutational spectrum between germline and sporadic *VHL *mutations may differ [25], which could explain the disparity. We were not able to select records for sporadic tumors in the database, but Gallou et al. [26] described mutations for 145 sporadic cases from this database. Compared to our results, the percentage of nonsense mutations was higher (8% vs. 3%) and the percentage of in-frame deletions or insertions was lower (4% vs. 12%) [26].

Furthermore, we searched the Universal *VHL *Mutation database [31] for the mutations observed in the cases with a positive family history of RCC. The T>A (silent) mutation at codon 139 (sample id 1003) was not reported before, the deletion of 1 nucleotide at codon 146 (sample id 306) was recorded in the database twice, while the second 1 nucleotide deletion at codon 167 (sample id 1600) had not been reported before.

For 10 RCC cases, we observed different *VHL *genotypes in different tumor samples obtained from one tumor. Samples were re-analyzed to exclude technical errors. Again, different *VHL *genotypes, identical to the earlier genotypes, were observed, showing that tumor heterogeneity appears to play a role. This was an unexpected finding in view of the general acceptance that a mutation in the *VHL *gene is an early event in clear-cell RCC carcinogenesis, because one would then expect monogenetic *VHL *expression. Nevertheless, it is possible that genetic instability leads to additional mutational events, eventually leading to different *VHL *genotypes in clear-cell RCC. This was underscored by 13 cases showing 2 mutations within the same paraffin block and other studies have also reported multiple mutations per primary tumor [21,27,29].

The distribution of histological subtypes was comparable to the distribution described before [[32], p2692]. Surprisingly, we also found *VHL *mutations in some of the non-clear-cell RCC samples that were collected. These results are in contrast with the current opinion that *VHL *mutations are exclusively restricted to clear-cell RCC. *VHL *mutations, however, have also been reported in chromophobe RCC, although these were clustered in the 5'UTR/promoter region [22], and Brauch et al. [12] described a renal oncocytoma with a *VHL *mutation. To our knowledge, others have not yet described *VHL *mutations in papillary RCC. It is conceivable that genetic instability following tumor-initiating events leads to *VHL *mutations acquired at a later stage of tumor development. As expected, the relative occurrence of *VHL *mutations in the non-clear-cell RCC renal tumors was much lower than in clear-cell RCC, emphasizing the crucial role of *VHL *mutations in clear-cell RCC carcinogenesis.

The high percentage of mutations observed, the observation of mutational heterogeneity (different genotypes in 1 or different samples from the same tumor), and the observation of mutations in other histological types, raises the question whether paraffin material is suitable for analysis. It has been shown, however, that *VHL *gene mutations can be detected in archival paraffin material [33]. Identical mutations were observed in exon 2 in 3 cases for which archival paraffin material and tumor derived cell lines were assessed by SSCP and sequencing [33]. On the other hand, it has also been reported that artifacts may occur when archival paraffin material is used [34]. Williams et al. reported up to 1 mutation artifact per 500 bases recorded. The chance of such artificial mutations in formalin-fixed material was inversely correlated with the number of cells used in the PCR: the fewer the cells, the more artifacts. No artifacts were observed when 300 cells were used and only 1 artifact was observed in 150 cells (0.03%). The highest frequency of artifacts was observed when using 10 or 20 cells, and equaled 0.2 % [34]. The number of cells in our analyses, however, was large (>500 cells), since 5 20-μm slices of paraffin embedded material were used and biopsies were excluded.

The mean tumor size was larger for mutated tumors than for wildtype tumors, however not statistically significant. Two other studies did not show a difference in tumor size [24,28]. *VHL *gene mutations leading to reduced or inactive VHL protein (pVHL) could theoretically lead to a larger tumor size. In the absence of pVHL, hypoxia inducible factor becomes stabilized and upregulates a myriad of hypoxia-inducible genes, resulting in hypervascular tumors [35]. This should be a growth advantage. Our results and results from others [24,28] showed no association with nuclear grade (according to Fuhrman) [19]. It was difficult to compare TNM and stage, since different classifications were used in different studies and the TNM classification has changed definitions in recent years. We reviewed all cases and classified them according to the 1987 version of the TNM classification [17]. We did not observe a difference in T, N, M, or stage between mutated and wildtype tumors, as was confirmed by 2 other studies [24,28]. One study, however, reported a higher percentage of mutations in pT3 tumors with a *VHL *mutation or hypermethylation, compared to tumors without *VHL *alteration (64% vs. 40%) [21].

Phenotype should also be considered. Mutations may impair pVHL function. However, in order for pVHL to lose its function, biallelic *VHL *defects have to occur. We did not measure LOH, since it is well documented that LOH commonly occurs in RCC cases, with estimates ranging from 74% [30] to 93% [21]. Kondo et al. showed 97% LOH in mutated or hypermethylated samples compared to 81% LOH in wildtype samples [24]. Furthermore, silent mutations do not lead to an altered protein, but these types of mutations were not common (13/131) in our study. Also, pVHL formation may be inhibited by epigenetic silencing, which has been reported to occur in 19% of clear-cell RCC [9]. This could have resulted in misclassification in comparing pathological parameters, because samples with epigenetic silencing only were classified as wildtype tumors, as we were not able to investigate epigenetic silencing in the current study. Finally, cells in cell culture have been shown to produce two types of proteins: pVHL30 and pVHL19 (a results of internal translation from the second methionine within the VHL open reading frame (Met 54) [36-38]. The pVHL19 product has been shown to inhibit the production of hypoxia-inducible genes, but does not bind to fibronectin [36,37]. Reintroduction of pVHL19 has been shown to inhibit tumor formation in nude mice [37]. We detected mutations 5' of codon 54 in 11 cases; these are unusual and would be predicted to affect pVHL30, but not pVHL19 translation products. However in 5 of these 11 cases, a second mutation was observed 3'of codon 54. So, in 5 of these cases the other mutation may be relevant. For the other 6 cases, these mutations may not lead to a functionally inactive protein, and thus we may have misclassified these. However, as remarked before, the pVHL19 variant does not bind to fibronectin and thus may not contribute to the maintenance of the extracellular matrix.

## Conclusion

*VHL *mutations were observed in 61% of sporadic clear-cell RCC. No differences were observed in nuclear grade, TNM distribution or stage between *VHL *mutated and wildtype clear-cell RCC. However, the tumor size was larger for *VHL *mutated clear-cell RCC. This study forms a good basis to study possible associations between potential risk factors and *VHL *mutations in clear-cell RCC. Ultimately, this should lead to greater insight in the etiology of clear-cell RCC.

## List of abbreviations

CI Confidence Interval

NLCS Netherlands Cohort Study on diet and cancer

RCC Renal Cell Carcinoma

RR Rate Ratio

VHL von Hippel-Lindau

## Competing interests

The author(s) declare that they have no competing interests.

## Authors' contributions

Paraffin embedded material was collected by BACD. KPH carried out laboratory analyses, aided by HJMG. CAHK revised all HE-stained slides from collected material. BACD carried out statistical analyses. KPH, BACD and EO co-drafted the document. CAHK, LJS, JAS and PAB revised the document critically for important intellectual content. PAB also initiated the Netherlands Cohort Study on diet and cancer, of which this study is a part. All authors read and approved the final manuscript.

## Pre-publication history

The pre-publication history for this paper can be accessed here:



## Supplementary Material

Additional File 1 Calculation of the estimated upper 95% confidence limit. Click here for file

Additional File 2 Description of observed mutations (N = 139), histological parameters and personal characteristics for 121 cases. Click here for file

## Figures and Tables

**Table 1:** Primers used to amplify the *VHL *gene

Name	Sequence 5'>3'	Exon	Tm	Fragment length
Sense3a	GGT CTG GAT CGC GGA GGG A	1	64°C *	191 bp
Asense4a	GCC CGG CCT CCA TCT CCT	1		
Sense5a	AGT CGG GCG CCG AGG AGT	1	64°C *	184 bp
Asense6a	CCG TCG AAG TTG AGC CAT AC	1		
Sense7a	CCC AGG TCA TCT TCT GCA AT	1	64°C †	159 bp
Asense8a	CTG CTG GGT CGG GCC TAA G	1		
Sense9	GTG GCT CTT TAA CAA CCT TTG C	2	60°C‡	194 bp
Asense10	CCT GTA CTT ACC ACA ACA ACC TTA TC	2		
Sense11a	CAC TGA GGA TTT GGT TTT TGC	3	55°C	162 bp
Asense12a	TCC AGG TCT TTC TGC ACA TTT	3		
Sense13a	GAC ATC GTC AGG TCG CTC TA	3	55°C	150 bp
Asense14a	TCA AAA GCT GAG ATG AAA CAG TG	3		

* 3% DMSO added for PCR

† 1.5% DMSO and 2.5 mM MgCl_2 _added for PCR

‡ 4% DMSO and 1.0 mM MgCl_2 _added for PCR

**Table 2:** Mutation analysis in 20 samples by SSCP and direct sequencing

		Direct sequencing	
		
		Positive	Negative
Primerset 3/4			
SSCP	Positive	0	5
	Negative	0	15
Primerset 5/6			
SSCP	Positive	2	0
	Negative	0	18
Primerset 7/8			
SSCP	Positive	4	2
	Negative	0	14
Primerset 9/10			
SSCP	Positive	3	0
	Negative	0	17
Primerset 11/12			
SSCP	Positive	4	1
	Negative	0	15
Primerset 13/14			
SSCP	Positive	2	0
	Negative	0	18

Positive on SSCP means: indication of a mutation or not assessable after two attempts.

Positive on direct sequencing means: proof of a mutation.

**Table 3:** Tumor parameters for patients with a clear-cell tumor, also stratified by VHL mutation status

	All tumors (N = 188)		VHL mutated tumors (N = 115)		Wildtype tumors (N = 73)			
					
	N	%	N	%	N	%	χ^2^	p-value †
T (TNM*)								
1	5	2.7	4	3.5	1	1.4		
2	93	49.5	55	47.8	38	52.1		
3A	35	18.6	20	17.4	15	20.6		
3B	50	26.6	34	29.6	16	21.9		
4	2	1.1	1	0.9	1	1.4	0.18§**	0.67
X	3	1.6	1	0.9	2	2.7		
N (TNM*)								
0	126	67.0	78	67.8	48	65.8		
1	7	3.7	6	5.2	1	1.4		
2	7	3.7	5	4.4	2	2.7	1.51¶**	0.22
X	48	25.5	26	22.6	22	30.1		
M (TNM*)								
0	131	69.7	77	67.0	54	74.0		
1	26	13.8	18	15.7	8	11.0	0.99**	0.32
X	31	16.5	20	17.4	11	15.1		
Stage (TNM*)								
1	5	2.7	4	3.5	1	1.4		
2	83	44.2	46	40.0	37	50.7		
3	66	35.1	42	36.5	24	32.9		
4	32	17.0	22	19.1	10	13.7	1.41§**	0.24
X	2	1.1	1	0.9	1	1.4		
Nuclear grading †‡								
I	46	24.5	27	23.5	19	26.0		
II	67	35.6	43	37.4	24	32.9		
III	48	25.5	29	25.2	19	26.0		
IV	27	14.4	16	13.9	11	15.1	0.43	0.94

* Based on pathological TNM unless unknown, then clinical TNM (UICC, 1987) was used [17]

† Based on the review by one experienced pathologist (CAHK)

‡ According to Fuhrman [19]

§1–2 vs. 3–4

¶1–2 vs. 0

** Excludes "X (unknown) " category
