# Supplementary material for: Prevalence of von Hippel-Lindau gene mutations in sporadic renal cell carcinoma: results from the Netherlands cohort study
Source: BMC Cancer. 2005 Jun 2;5:57. doi: 10.1186/1471-2407-5-57 (PMC1177929; doi:10.1186/1471-2407-5-57)
Supplement: Additional File 1 — Calculation of the estimated upper 95% confidence limit. [file 1471-2407-5-57-S1.doc]

# Additional file 1 – Calculation of the estimated upper 95% confidence limit

The point estimate for the percentage of samples negative on the SSCP and positive on direct sequencing equals 0% (Table 2); the upper confidence limit was calculated by the following formula: P(X=0 | N, Pu) =0.025 → (1-Pu)^N^ = 0.025. This analysis was performed for two situations. Situation 1; calculation of upper 95% confidence limit based on the cases and situation 2; calculation of upper 95% confidence limit based on the individual analyses. The N differs for these situations described; for situation 1, N equals 20 (there are 20 cases for with at least one negative SSCP, followed by a negative result on direct sequencing), for situation 2 N equals 97 (of all 120 SSCP results, 97 were negative and were followed by a negative result on direct sequencing). The estimations of the upper confidence limit are: based on situation 1: P(x=0|N=20, P=Pu)=0.025 → (1-Pu)^20^=0.025 → Pu=0.1685 and based on situation 2: P(x=0|N=97, P=Pu)=0.025 → (1-Pu)^97^=0.025 → Pu=0.0373. However, both of these estimates are not completely true, because in the first situation we assume all the analyses on all primer sets to be dependent and for the second estimation, we assume all the analyses of all primer sets to be completely independent, neither of which is true. Primer sets are not completely independent, since some primer sets were selected to overlap to sequence the whole coding sequence of the gene. In total, 204 out of 1042 nucleotides were determined by more than 1 primer set. Also, the quality of the extracted DNA and other properties of the DNA may render the analyses by different primer sets not independent.
