# Supplementary material for: Prevalence of von Hippel-Lindau gene mutations in sporadic renal cell carcinoma: results from the Netherlands cohort study
Source: BMC Cancer. 2005 Jun 2;5:57. doi: 10.1186/1471-2407-5-57 (PMC1177929; doi:10.1186/1471-2407-5-57)
Supplement: Additional File 2 — Description of observed mutations (N = 139), histological parameters and personal characteristics for 121 cases. [file 1471-2407-5-57-S2.doc]

## Additional file 2 - Description of observed mutations (N=139), histological parameters and personal characteristics for 121 cases

| Sample | Sex /  Age at incidence | TNM classification* | | | Stage * | Histology† | Nuclear Grade  †‡ | % Vital tumor tissue † | VHL status  Mutation § |
| --- | --- | --- | --- | --- | --- | --- | --- | --- | --- |
|  |  | T | N | M |  |  |  |  |  |
| 941 | M / 71 | 2 | 0 | 0 | II | Clear-cell | I | 75 | [c.1-1_20del21 (+)  c.291_310del20] |
| 349 | F / 68 | 2 | 0 | 0 | II | Clear-cell | III | 50 | c.102C>T |
| 2048 | M / 65 | 3B | 0 | 0 | III | Clear-cell | IV | 85 | c.113C>T |
| 1782 | M / 66 | 3B | 2 | X | IV | Clear-cell | III | 90 | [c.115G>A (+)  c.291C>A] |
| 1716 | F / 58 | 2 | X | X | II | Clear-cell | II | 50 | [c.118C>T (+)  c.145G>T] |
| 973  974 | M / 61 | 3B | 0 | 0 | III | Clear-cell | III  III | 60  90 | [c.122A>T (+)  c.183C>T] |
| 1013 | F / 70 | 2 | 1 | 1 | IV | Clear-cell | III | 100 | c.125A>T |
| 187 | M / 74 | 3B | 0 | 0 | III | Clear-cell | II | 100 | c.142C>G |
| 336 | M / 65 | 2 | 0 | 0 | II | Clear-cell | II | 90 | [c.149C>G (+)  c.221T>C] |
| 1524 1525 | M / 64 | 2 | 0 | X | II | Clear-cell | II  II | 100  100 | [c.151G>C (+)  c.284C>G] |
| 463 | F / 66 | 3B | 0 | 0 | III | Clear-cell | III | 100 | c.158ins45 |
| 1510 | F / 68 | 2 | 0 | 0 | II | Clear-cell | I | 100 | c.166del1 |
| 2109 | F / 65 | 2 | 0 | 0 | II | Clear-cell | I | 100 | [c. 183del1 (+)  c. 463+5T>A] |
| 191 | F / 66 | 2 | 0 | 0 | II | Clear-cell | I | 90 | c.183_187del5 |
| 683 | M / 74 | 3A | 0 | 0 | III | Clear-cell | II | 100 | c.183_187del5 |
| 1306 | M / 62 | 3A | 0 | 0 | III | Clear-cell | II | 100 | c.183C>T |
| 260 | M / 69 | 2 | 0 | 0 | II | Clear-cell | I | 100 | c.183ins5 |
| 14 | M / 66 | 2 | 0 | 0 | II | Clear-cell | II | 100 | c.185ins5 |
| 669 | M / 65 | 3B | 0 | 0 | III | Clear-cell | IV | 70 | c.195G>C |
| 829 | M / 66 | 3A | 0 | 0 | III | Clear-cell | III | 60 | c.198_221del24 |
| 592 | M / 73 | 2 | 0 | 0 | II | Clear-cell | III | 100 | [c.202T>A (+)  c.203C>A] |
| 1809 | F / 65 | 2 | 0 | 0 | II | Clear-cell | II | 90 | [c.204del1 (+)  c.283_297del15] |
| 2002 | F / 65 | 3B | 0 | 0 | III | Clear-cell | III | 90 | c.205del1 |
| 757 | M / 67 | 3B | 1 | X | III | Clear-cell | I | 95 | c.208G>T |
| 2043 | M / 70 | 3A | 0 | 0 | III | Clear-cell | III | 70 | c.215_259del45 |
| 2438 | M / 77 | 3B | 0 | 1 | IV | Clear-cell | II | 50 | c.217del1 |
| 2035 | M / 72 | 2 | 0 | 1 | IV | Clear-cell | I | 90 | c.219G>A |
| 2049 | M / 68 | 3A | X | X | III | Clear-cell | IV | 90 | c.221T>A |
| 597 | F / 71 | 2 | 0 | 0 | II | Clear-cell | I | 95 | c.227del1 |
| 1323 | M / 58 | 4 | X | 1 | IV | Clear-cell | IV | 100 | c.231_232del2 |
| 593 | M / 70 | 2 | 0 | 0 | II | Clear-cell | II | 95 | c.231C>A |
| 334 | M / 70 | 3B | X | 0 | III | Clear-cell | III | 80 | c.234_237del4 |
| 562 | M / 65 | 3B | 0 | 0 | III | Clear-cell | II | 100 | c.236_237del2 |
| 1798 | M / 67 | 2 | X | X | II | Clear-cell | III | 95 | c.239_241del3 |
| 926 | M / 73 | 3B | 1 | 0 | III | Clear-cell | II | 70 | c.240T>A |
| 949 | M / 60 | 3B | 2 | X | IV | Clear-cell | III | 50 | c.241C>T |
| 879 | M / 70 | 2 | 0 | X | II | Clear-cell | III | 48 | c.253ins23 |
| 1502 | M / 65 | 2 | 0 | 1 | IV | Clear-cell | III | 100 | c.268_270del3 |
| 1651 | F / 58 | 2 | 0 | 0 | II | Clear-cell | III | 100 | c.268_274del7 |
| 6 | M / 64 | 2 | 0 | 0 | II | Clear-cell | II | 100 | c.270_272del3 |
| 7 | M / 64 | 3A | 1 | 1 | IV | Clear-cell | III | 50 | c.274G>T |
| 424 | F / 69 | 2 | 0 | X | II | Clear-cell | I | 100 | c.282ins1 |
| 229 | M / 75 | 2 | 0 | 0 | II | Clear-cell | IV | 45 | c.288_291del4 |
| 2088 | F / 70 | 2 | 0 | 0 | II | Clear-cell | II | 67 | c.288G>T |
| 1339 | F / 68 | 3B | 0 | 0 | III | Clear-cell | III | 50 | c.291C>T |
| 755 | M / 67 | 3B | X | 0 | III | Clear-cell | II | 95 | c.301C>T |
| 1342 | F / 74 | 2 | 0 | 0 | II | Clear-cell | II | 90 | c.302_304del3 |
| 176 | M / 69 | 3B | X | X | III | Clear-cell | II | 100 | [c.304C>G (+)  c.444del1] |
| 695 | F / 69 | 3B | 0 | 0 | III | Clear-cell | II | 90 | c.306_308del3 |
| 343 | F / 60 | 2 | 0 | 0 | II | Clear-cell | III | 70 | c.314C>T |
| 954 | M / 70 | 3A | X | X | III | Clear-cell | IV | 100 | [c.321_323del3 (+)  c.426T>G] |
| 161 | M / 69 | 2 | 0 | 0 | II | Clear-cell | I | 66 | c.324C>A |
| 1350 | F / 65 | 1 | 0 | 0 | I | Clear-cell | I | 85 | c.324del1 |
| 965 | M / 67 | 3B | X | X | III | Clear-cell | I | 90 | c.328_333del6 |
| 49 | F / 66 | 3B | X | 1 | IV | Clear-cell | II | 90 | [c.330del1 (+)  c.381del1] |
| 307 | M / 63 | 2 | X | X | II | Clear-cell | II | 95 | c.331del1 |
| 48 | M / 63 | 3B | 0 | 0 | III | Clear-cell | IV | 100 | c.333del1 |
| 189 | M / 58 | 3B | 0 | 0 | III | Clear-cell | III | 95 | c.334T>G |
| 1912 | M / 66 | 3A | 0 | 0 | III | Clear-cell | III | 100 | c.339A>T |
| 904 | F / 65 | 2 | 1 | 0 | III | Clear-cell | II | 100 | c.340G>T |
| 38 | F / 66 | 3A | 0 | 0 | III | Clear-cell | II | 100 | [c. 340G>A (+)  c. 340+2T>G] |
| 2458 | F / 57 | 3A | X | 0 | III | Clear-cell | I | 85 | c.340+9C>T |
| 262 | F / 64 | 2 | 0 | 0 | II | Clear-cell | I | 90 | c.341-2del1 |
| 690 | F / 75 | 3B | X | 1 | IV | Clear-cell | III | 60 | c.341-1_367del28 |
| 1299 | M / 72 | 2 | 0 | 0 | II | Clear-cell | II | 90 | c.343C>A |
| 596 | F / 66 | 2 | 0 | 0 | II | Clear-cell | III | 100 | c.348_354del7 |
| 338 | M / 68 | 3B | 0 | 0 | III | Clear-cell | III | 100 | c.353T>A |
| 9 | M / 70 | 3A | X | 1 | IV | Clear-cell | IV | 100 | c.373_378del6 |
| 400 | F / 73 | 2 | 0 | X | II | Clear-cell | II | 90 | c.383T>A |
| 2057 | M / 68 | 2 | X | X | II | Clear-cell | IV | 50 | c.390del1 |
| 2451 | F / 66 | 2 | 0 | 1 | IV | Clear-cell | II | 95 | c.391A>T |
| 1167 | M / 74 | 1 | 0 | 0 | I | Clear-cell | I | 30 | c.393_396del4 |
| 2446 | M / 72 | 3B | 1 | X | III | Clear-cell | III | 85 | c.393_396del4 |
| 559 | M / 67 | 3A | 0 | 0 | III | Clear-cell | I | 100 | c.406_408del3 |
| 854 | M / 65 | 3A | 0 | 0 | III | Clear-cell | III | 70 | c.406ins1 |
| 2078 | M / 71 | 3A | 0 | 1 | IV | Clear-cell | III | 80 | c.408del1 |
| 777 | M / 67 | 3B | 0 | 0 | III | Clear-cell | II | 100 | c.408T>G |
| 309 | M / 63 | 3A | 0 | X | III | Clear-cell | I | 100 | c.409del1 |
| 2449 | F / 75 | 2 | 0 | 0 | II | Clear-cell | II | 100 | c.413_414del2 |
| 947 | M / 80 | X | 0 | 0 | X | Clear-cell | II | 100 | c.417T>A |
| 1003 | F / 70 | 1 | 0 | 0 | I | Clear-cell | I | 60 | c.417T>A |
| 670 | M / 77 | 3B | 2 | 0 | IV | Clear-cell | I | 60 | c.418_425del8 |
| 461 | F / 65 | 3A | 0 | 0 | III | Clear-cell | I | 100 | c.431del1 |
| 306  1600 | M / 67 | 3B | 2 | 0 | IV | Clear-cell | IV  IV | 75  75 | [c.437del1 (+)  c.501del1] |
| 651 | F / 71 | 3A | X | 1 | IV | Clear-cell | IV | 70 | c.457_463del7 |
| 1334 | F / 75 | 2 | 0 | 1 | IV | Clear-cell | II | 100 | c.458ins4 |
| 952 | M / 72 | 2 | 0 | 1 | IV | Clear-cell | IV | 100 | c.462ins1 |
| 398 | F / 70 | 2 | X | 0 | II | Clear-cell | IV | 100 | c.463+3A>T |
| 2092 | F / 62 | 3B | 2 | 1 | IV | Clear-cell | IV | 80 | c.463+8C>T |
| 989 | M / 66 | 2 | 0 | 0 | II | Clear-cell | I | 90 | c.463+23A>G |
| 1325 | M / 69 | 2 | X | 0 | II | Clear-cell | I | 85 | c.464-2_469del8 |
| 2455 | M / 71 | 2 | 0 | 0 | II | Clear-cell | II | 70 | c.464-1G>A |
| 1332 | M / 66 | 2 | 0 | X | II | Clear-cell | II | 90 | c.464-1G>C |
| 1303 | M / 66 | 2 | 0 | 0 | II | Clear-cell | II | 70 | c.472C>G |
| 682 | M / 75 | 2 | X | 0 | II | Clear-cell | III | 95 | [c.480del1 (+)  c.482_483del2] |
| 170 | F / 67 | 2 | 0 | 0 | II | Clear-cell | II | 67 | c.481_483del3 |
| 692 | F / 70 | 2 | X | 0 | II | Clear-cell | I | 85 | c.481C>T |
| 2127 | F / 74 | 3B | 0 | X | III | Clear-cell | IV | 90 | c.482ins1 |
| 960 | M / 60 | 2 | 0 | 0 | II | Clear-cell | II | 90 | c.487del1 |
| 1329 | M / 73 | 2 | 0 | 1 | IV | Clear-cell | II | 95 | c.494T>A |
| 944 | M / 69 | 2 | 0 | 0 | II | Clear-cell | III | 100 | c.497T>G |
| 568 | F / 76 | 3A | X | 0 | III | Clear-cell | I | 50 | c.523del1 |
| 1314 | M / 66 | 3B | 0 | 0 | III | Clear-cell | II | 50 | c.525_532del8 |
| 1170 | M / 66 | 1 | 0 | 0 | I | Clear-cell | II | 80 | c.525C>A |
| 1297 | M / 59 | 3A | X | 1 | IV | Clear-cell | IV | 100 | c.529del1 |
| 787 | M / 70 | 3A | 0 | 0 | III | Clear-cell | III | 50 | c.529ins1 |
| 11 | M / 69 | 2 | X | 0 | II | Clear-cell | II | 100 | c.547del1 |
| 506 | F / 76 | 2 | 0 | 0 | II | Clear-cell | II | 80 | [c.559_560del2 (+)  c.563del1] |
| 496  497 | M / 66 | 3B (2) | 0 | 0 | III | Clear-cell | I  II | 100  100 | [c.559_560del2 (+)  c.561_563del3] |
| 1001 | F / 61 | 2 | X | X | II | Clear-cell | II | 100 | c.561_564del4 |
| 1 | M / 60 | 3B | 0 | 0 | III | Clear-cell | II | 100 | c.563T>A |
| 369 | M / 74 | 2 | X | 1 | IV | Clear-cell | I | 80 | c.566_569del4 |
| 1004 | F / 69 | 2 | 0 | 0 | II | Clear-cell | I | 100 | c.567del1 |
| 2086 | F / 74 | 3B | X | 0 | III | Clear-cell | II | 100 | c.573_577del5 |
| 962  963 | M / 74 | 3B | 0 | 0 | III | Chromophobe | IV  IV | 50  50 | [c.1_17del17 (+)  c.471T>A] |
| 347 | M / 67 | 3A | 0 | 0 | III | Chromophobe | II | 40 | c.333C>T |
| 1016 | F / 65 | 2 | X | X | II | Oncocytoma | X | 90 | c.113C>T |
| 1499 | M / 69 | 2 | 0 | 0 | II | Papillary | II | 100 | c.264G>C |
| 31 | M / 63 | 2 | 0 | X | II | Papillary | III | 100 | c.443T>C |
| 2456 | F / 73 | 2 | 3 | 0 | IV | Unclassified | IV | 80 | c.245G>C |
| 16 | F / 65 | 3A | 0 | 0 | III | Unclassified | IV | 90 | c.353T>C |

*Based on pathological TNM unless unknown, then clinical TNM (UICC, 1987) was used [1]

†Based on the review by one experienced pathologist (CAHK)

‡According to Fuhrman [2]

§ The notation of von Hippel-Lindau gene mutations was based on the guidelines described at the following website: www.genomic.unimelb.edu.au/mdi/mutnomen/examplesDNA.html (Exon 1: c.1 through c.340; exon 2: c.341 through c.463; exon 3: c464 through c.642)

1. Hermanek P, Sobin L: **TNM Classification of Malignant Tumours**, Fourth, fully revised edition edn: Springer-Verlag; 1987.

2. Fuhrman S, Lasky L, Limas C: **Prognostic significance of morphologic parameters in renal cell carcinoma**. *Am J Surg Pathol* 1982, **6**:655-663.
